# Optimal predictor for 6-mercaptopurine intolerance in Chinese children with acute lymphoblastic leukemia: NUDT15, TPMT, or ITPA genetic variants?

**DOI:** 10.1186/s12885-018-4398-2

**Published:** 2018-05-02

**Authors:** Hong Zhou, Lei Li, Peng Yang, Lin Yang, Jin E. Zheng, Ying Zhou, Yong Han

**Affiliations:** 1 Department of Pharmacy, Union Hospital, Tongji Medical College, Huazhong, University of Science and Technology, No. 1277, Jie Fang Road, Wuhan, 430022 China; 2 Department of Pediatrics, Union Hospital, Tongji Medical College, Huazhong, University of Science and Technology, Wuhan, 430022 China; 3 Department of Geriatrics, Union Hospital, Tongji Medical College, Huazhong University of Science and Technology, Wuhan, 430022 China; 4 Department of Pharmacy, Fujian Cancer Hospital & Fujian Medical University Cancer Hospital, Fuzhou, 350014 China; 5 Center for Stem Cell Research and Application, Union Hospital, Tongji Medical College, Huazhong University of Science and Technology, Wuhan, 430022 China

**Keywords:** NUDT15, 6-mercaptopurine, Tolerance, Acute lymphoblastic leukemia (ALL)

## Abstract

**Background:**

6-mercaptopurine (6-MP) contributes substantially to remarkable improvement in the survival of childhood acute lymphoblastic leukemia (ALL) patients. However, 6-MP also has dose-limiting toxicities, particularly life-threatening myelosuppression, due to genetic polymorphisms in enzymes that metabolize 6-MP. Promising biomarkers for predicting 6-MP-induced leukopenia is still unclear in Chinese population. Here, we evaluated the associations of NUDT15, TPMT and ITPA genotypes with 6-MP intolerance in our cohort of childhood ALL patients.

**Methods:**

A total of 105 Chinese pediatric patients with a confirmed diagnosis of ALL were enrolled. We identified the NUDT15 coding variant rs116855232 (c.415C > T), a newly discovered 6-MP toxicity-related locus in Asians, and polymorphisms in TPMT rs1142345 and ITPA rs11273540. Associations between genotypes and 6-MP dose sensitivity, leukopenia, hepatotoxicity, and therapy interruption were evaluated.

**Results:**

The minor allele frequencies (MAFs) of NUDT15 rs116855232, TPMT rs1142345 and ITPA rs11273540 were 15.7, 2.9, and 18.1%, respectively. NUDT15 and TPMT genetic variants were strongly associated with 6-MP dose intensity. Patients with NUDT15 homogenous genotype (TT) were highly sensitive to 6-MP (dose intensity of 60.27%) compared to these with heterozygous genotype (TC) or wild type (CC), who tolerated an average dose intensity of 83.83 and 94.24%, respectively. The NUDT15 variant was a predictor for leukopenia (OR: 3.62, 95% CI 1.377–9.501, *P* = 0.009) and early-onset leukopenia (OR: 9.63, 95% CI 2.764–33.514, *P* = 3.75 × 10^− 4^). No differences were found between 6-MP dose intensity and ITPA polymorphisms.

**Conclusion:**

NUDT15 variant is an optimal predictor for 6-MP intolerance in Chinese pediatric ALL patients and may have greatly clinical implications for individualized therapy.

## Background

Acute lymphoblastic leukemia (ALL) is responsible for almost a third of all childhood cancers and can be cured with combination chemotherapy alone [1–3]. 6-mercaptopurine (6-MP) is one of the most commonly prescribed chemotherapeutic agents to treat ALL [4–6]. Despite the acknowledged efficacy of 6-MP in ALL, treatment of this disease remains challenging due to the considerable variability in toxicity among patients, especially life-threatening leukopenia [4, 7]. This severe toxicity usually results in interruption or even discontinuation of potentially effective anticancer therapy, contributing to an increased incidence of late relapse. Leukopenia also leads to a high risk of infection and often requires hospitalization, increasing health care costs. 6-MP intolerance is mostly associated with a deficiency in the activity of the enzymes thiopurine S-methyltransferase (TPMT) [8] and inosine triphosphate pyrophosphohydrolase (ITPA) [9], which are related to the metabolism of 6-MP, and presents wide inter-individual variability, partly arising from genetic polymorphisms.

Currently, pharmacogenetic association studies between TPMT single nucleotide polymorphisms (SNPs) and 6-MP tolerance have mainly focused on four variant alleles (TPMT*3A, TPMT*3C, TPMT*2 and TPMT*3B) [10]. TPMT*3A is very common in Caucasians, while TPMT*3C is common in Asians and Africans [11]. TPMT genotyping is a successful example of pharmacogenetic implementation in clinical practice. The Clinical Pharmacogenetics Implementation Consortium (CPIC) released evidence based guidelines for upfront TPMT genotyping to individualize thiopurine therapy [12]. TPMT testing before starting thiopurine drugs is also recommended by the British National Formulary [13]. Although loss of function of TPMT is a robust predictor of thiopurine-induced leukopenia, quite a few patients who are wild type for TPMT still develop toxicity that requires 6-MP dose reduction or treatment interruption [14]. Moreover, the frequency of the TPMT polymorphism is considerably lower in Asians, with the lowest frequencies of observed in Chinese (approximately 2.2%) [15, 16], than in populations of European descent [17]. The low population frequencies of these variants resulted in a lack of sensitivity in some studies. Paradoxically, East Asian patients are more sensitive to full dose of 6-MP [18], suggesting that additional variables, including other genetic variants, may contribute to the inter-patient variability in 6-MP-induced leukopenia.

ITPA deficiency in patients receiving 6-MP leads to toxic accumulation of 6-thioinosine triphosphate (6-TITP). The most well established deleterious ITPA variants associated with 6-MP-associated toxicity are rs1127354 (c.94C > A) and rs7270101 (IVS2 + 21A > C) [19, 20]. ITPA enzyme deficiency arising from genetic variants affects 5–7% of Caucasians and Africans, and up to 15% of Asians [11]. Despite associations between ITPA deficiency and 6-MP -related adverse effects, subsequent studies, both retrospective [21] and prospective [22], have been unable to replicate these findings. Similarly, no association between ITPA c.94C > A polymorphism and thiopurine-induced toxicity was found in patients with inflammatory bowel disease (IBD) [23]. Consequently, the clinical relevance of 6-MP-toxicity prediction from ITPA genotyping is still controversial and currently not recommended in clinical practice.

More recently, genome-wide association studies (GWAS) demonstrated that a missense variant (rs116855232, c.415C > T) in the nucleoside diphosphate-linked moiety X motif 15 (NUDT15) gene is strongly associated with thiopurine-related hematopoietic toxicity in patients with IBD [24] and in children with ALL [14]. In particular, the NUDT15 variant was most common in East Asians and Hispanics, rare in Europeans, and not observed in Africans, contributing to ethnic differences in 6-MP tolerance [14]. The incidence of severe myelotoxicity was more frequent in patients with NUDT15 CT or TT genotype, when receiving standard-dose 6-MP therapy, as replicated by other studies [21, 25–27]. These results indicate potentially clinical implications of NUDT15 genotyping and comprehensive pharmacogenetic models integrating NUDT15 variants to individualize 6-MP therapy. The prevalence of the NUDT15 variant and these inspiring associations in Chinese ALL patients, however, are still unknown.

Thus, the primary objective of this observational study was to identify the NUDT15 frequency in Chinese ALL patients and to confirm the association between the deleterious variant NUDT15 c.415C > T and 6-MP sensitivity and toxicity. Furthermore, in an exploratory analysis, genetic variants in TPMT, ITPA, and NUDT15 were compared and screened to determine the optimal risk prediction of 6-MP-induced leukopenia.

## Methods

### Patient recruitment and 6-MP treatment

Children with standard-risk and intermediate-risk ALL at Union Hospital, Tongji Medical College, Huazhong University of Science and Technology were enrolled in this study. Patients with high-risk ALL were excluded due to the different 6-MP maintenance protocol. All of the patients received maintenance therapy for at least 6 months according to the Chinese Children’s Leukemia Group (CCLG) protocol-ALL 2008 [28, 29] between 2013 and 2015. The medications administered during the maintenance phase consisted of monthly intravenous vincristine (VCR), a monthly pulse of dexamethasone (DEX), weekly oral methotrexate (MTX), daily oral 6-MP and intrathecal MTX once every two months. The initial doses of 6-MP and MTX for maintenance therapy were 50 mg/m^2^ daily and 20 mg/m^2^ weekly, respectively. A complete blood count was performed at a 4-week interval. 6-MP was either increased or decreased by 50% of the previous dose or even discontinued to maintain a white blood cell (WBC) count of 2.0–3.0 × 10^9^/L and/or avoid occurrence of infections and hepatotoxicity. 6-MP dose intensity was defined as the ratio between clinician prescribed 6-MP dose and protocol dose (%) and was captured on a monthly basis for the 6-month duration of the study [14].

Interruption was defined as the cessation of the administration of medicine resulting from infections and/or hepatotoxicity. Leukopenia (WBC < 2.0 × 10^9^/L) was based on the Common Terminology Criteria for Adverse Events version 4.0 (CTCAE4.0), and hepatotoxicity was defined as an ALT or AST level > 500 U/L at any time point during maintenance therapy. Early-onset leukopenia was defined as leukopenia occurrence during the first 60 days of the maintenance therapy.

The study was conducted with the approval of the institutional ethics committee. Written informed consent was obtained from the parents or guardians of the patients or from the patients themselves, depending on the age and conceptual ability of the patients.

### Genetic analyses

Genomic DNA was extracted from 200 μl EDTA-treated peripheral blood according to the instructions of the QIAamp DNA Blood Mini Kit (Qiagen, Hilden, Germany). DNA was stored at − 80 °C for further detection, after quantification using a spectrophotometer (Thermo, Inc., DE, USA) to determine the concentration and purity. NUDT15 (c.415C > T, rs116855232, p.Arg139Cys), NUDT15 (c.52G > A, rs186364861), TPMT*3C (719 A > G, rs1142345, Tyr240Cys), TPMT*2 (238G > C, rs1800462, Ala80Pro), ITPA (c.94 C > A, rs1127354, p.Pro32Thr), and ITPA (IVS2 + 21A > C, rs7270101) were genotyped by Beijing Genomics Institute (BGI, Shenzhen, China) using the Mass ARRAY platform (Sequenom, San Diego, CA, USA). All samples were analyzed in triplicate, and both negative and positive controls were included to ensure the authenticity of the results.

### Statistical analysis

Statistical analysis and calculations were conducted using the SPSS software version 19.0 (IBM, Armonk, NY, USA) and Prism 5 (Graph Pad software, La Jolla, CA, USA). Clinical data were presented as means and standard deviation (SD) or as absolute frequencies and percentages as appropriate. Quantitative variables were expressed as median and range. Categorical variables, such as the incidence of leukopenia or therapy interruption were expressed as proportions and compared using the χ^2^ test or Fisher’s test if the number of subjects in any cell of 2 × 2 table was five or less. 6-MP dose intensity was summarized as medians with SDs, and comparisons among NUDT15, TPMT, and ITPA genotypes were performed using the Mann-Whitney U test (*n* = 2) or Kruskal-Wallis test (*n* = 3). All genotype frequencies were computed and tested for Hardy–Weinberg equilibrium with the χ^2^ test. Odds ratios and 95% confidence intervals were determined using logistic regression analysis. Receiver operating characteristic (ROC) curves were obtained to plot the sensitivity and specificity for NUDT15 genotypes to predict the development of leukopenia. A two-sided *P* value of less than 0.05 was considered statistically significant.

## Results

### Genotype frequencies

In the present study, six SNPs in three genes were analyzed in 105 children with ALL. Surprisingly, no genetic polymorphisms in NUDT15 (c.52G > A, rs186364861), TPMT*2 (238G > C, rs1800462), or ITPA (IVS2 + 21A > C, rs7270101) were observed. Of all the 105 patients, 74 were wild type (CC), 29 were heterozygous (CT) and only two were NUDT15 homozygous (TT) with an overall risk allele frequency of 15.7%. Patients’ characteristics based on NUDT15 were shown in Table 1. Only six patients carried the risk allele of TPMT rs1142345 with an overall risk allele frequency of 2.9%. Regarding ITPA, thirty-seven patients carried at least one ITPA A allele (only one patient was ITPA homozygous) with a frequency of 18.1%. The frequency of these genotypes and alleles did not deviate from Hardy–Weinberg equilibrium (*P* > 0.05).

**Table 1:** Characteristics of patients with ALL according to NUDT15 genotype

		NUDT15 (rs116855232, c.415C > T)		
	Total patients	CC	CT	TT
		74	29	2
Sex				
Male	66 (62.9%)	48	16	2
Female	39 (37.1%)	26	13	0
Age(/year)	5.8 (1.1~ 14.0)	5.8 (1.0~ 14.0)	5.5 (2.0~ 14.0)	6.8 (4.0~ 9.0)
BSA(/m^2^)	0.82 (0.48~ 1.53)	0.81 (0.48~ 1.33)	0.79 (0.49~ 1.53)	1.33 (1.21~ 1.46)
Risk group				
standard-risk	67 (63.8%)	46	19	2
median-risk	38 (36.2%)	28	10	0
Immunologic subtype				
B cell	101	73	26	2
T cell	4	1	3	0
TPMT 719 A > G				
*1/*1	99	69	30	0
*1/*3C	6	5	1	0
ITPA 94C > A				
CC	68	49	17	2
CA	36	24	12	0
AA	1	1	0	0

### Associations between genetic variants and 6-MP sensitivity

**Fig. 1:**
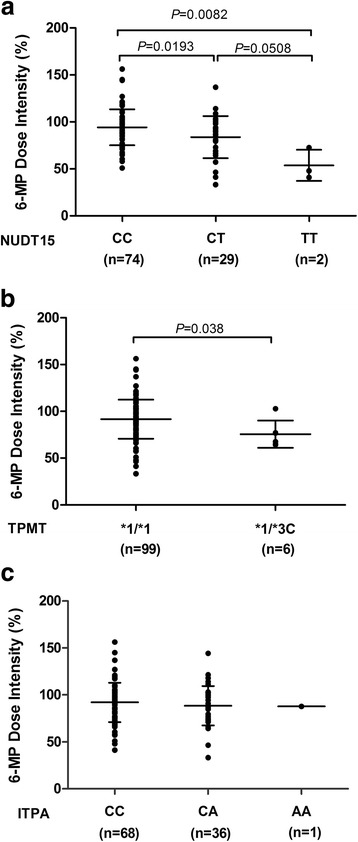
6-MP dose intensity and genotype of NUDT15 (**a**), TPMT (**b**), and ITPA (**c**). Patients with ALL received maintenance therapy with 50 mg/m^2^/d 6-MP according to the CCLG -ALL 2008 protocol. 6-MP dose intensity was defined as the ratio between clinician-prescribed 6-MP dose and protocol dose (%) and was captured on a monthly basis for the 6-month duration of the study. *P* values were estimated using Mann–Whitney or Kruskal–Wallis nonparametric testing for comparison of independent samples as applicable

Based on the CCLG -ALL 2008 protocol, 6-MP dose was optimized according to toxicities and/or infections during maintenance therapy. Therefore, 6-MP dose intensity, defined as the ratio of the prescribed 6-MP dose over the protocol dose of 50 mg/m^2^/d, directly reflected drug sensitivity/tolerance. In the present study, we observed that two genetic variants were significantly associated with 6-MP dose intensity: rs116855232 in NUDT15 (c.415C > T, *P* = 0.018; Fig. 1a) and rs1142345 in TPMT (719A > G, *P* = 0.038; Fig. 1b). Only two patients were homozygous for NUDT15 c.415C > T, and these individuals were highly sensitive to 6-MP, with a dose intensity of 60.27%, compared with these with the heterozygous genotype (*n* = 29) or wild type (*n* = 74), who tolerated an average dose intensity of 83.83 and 94.24%, respectively. However, no patients in our cohort were homozygous for C allele at TPMT rs1142345. As expected, patients carrying the TC genotype of TPMT (*n* = 6) were also sensitive to 6-MP with an average of 75.54% of the protocol standard dose. The polymorphism at ITPA rs1127354 (c.94 C > A) was not associated with 6-MP intensity in maintenance therapy (*P* = 0.583; Fig. 1c).

**Fig. 2:**
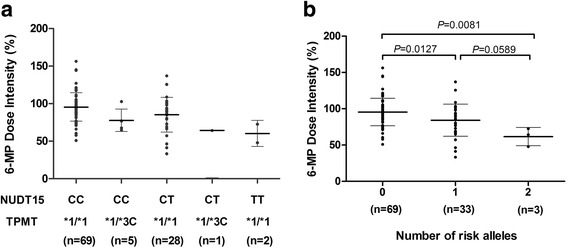
Combined effects of NUDT15 and TPMT on 6-MP dose intensity. **a** Patients were classified based on NUDT15 and TPMT genotypes. **b** Patients were classified as 0, 1 and 2 according to the number of risk alleles in NUDT15 and TPMT. A strong correlation was shown between the burden of risk alleles and dose intensity (*P* = 0.0049)

To further evaluate the mixed effects of both TPMT and NUDT15 variants on 6-MP intolerance, we compared 6-MP dose intensity in patients with different genotype combinations at these two loci. As previously reported [14], we assigned each patient a genetic risk score based on the number of risk alleles in both NUDT15 and TPMT. The burden of risk alleles were strongly correlated with 6-MP dose intensity (*P* = 0.0049), with patients carried NUDT15 TT genotype (*n* = 2) having the lowest 6-MP dose intensity (Fig. 2a). Patients with one risk allele at both TPMT and NUDT15 variants (*n* = 1) had similar 6-MP dose intensity to those with homozygous NUDT15 genotype. Children with heterozygous genotype for either TPMT or NUDT15 risk allele showed a trend toward a slightly increased 6-MP dose intensity, but with no statistical significance, compared with those homozygous for NUDT15 variant or heterozygous for both TPMT and NUDT15 gene(*P* = 0.0589). Associations between 6-MP dose intensity and the number of risk alleles were presented in Fig. 2b.

### Association of NUDT15 genotype with chemotherapy toxicity

**Fig. 3:**
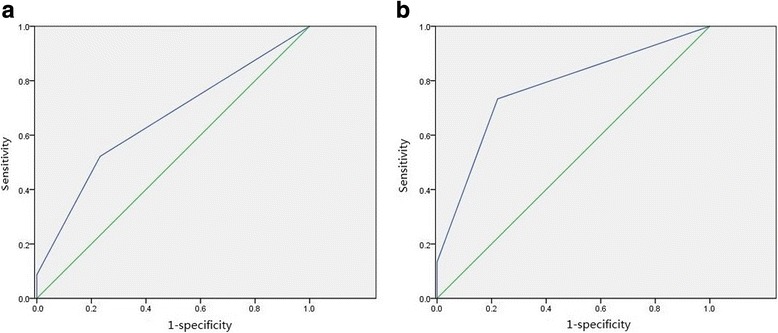
ROC curve for different additive prediction models of leukopenia and early-onset leukopenia using NUDT15 c.415C > T. The sensitivity of predictability of leukopenia (**a**) was 52.17% (12/23) with an AUC value of 0.655. The sensitivity was 73.33% (11/15) for predicting early early-onset leukopenia (**b**) with an AUC value of 0.770

In total, 6-MP-induced leukopenia was observed in 23 patients during the first 6 months of maintenance therapy. Leukopenia was more frequent in NUDT15 T allele carriers (CT + TT) and was associated with a 3.62-fold increased risk compared with that in patients with wild type (*P* = 0.009) (OR, 3.62; 95% CI, 1.377–9.501; Table 2). The NUDT15 c.415C > T had a sensitivity of 52.17% (12/23) and specificity of 76.83% (63/82) for predicting leukopenia, with an area under the curve (AUC) of 0.655 (Fig. 3a). Moreover, early-onset leukopenia was also remarkably associated with carriers of NUDT15 risk genetic variants (*P* = 3.75 × 10^− 4^) (OR, 9.63; 95% CI, 2.764–33.514; Table 2). The NUDT15 c.415C > T had a sensitivity of 73.33% (11/15) and specificity of 77.78% (70/90) for predicting early-onset leukopenia, with an AUC of 0.770 (Fig. 3b). In contrast, 6-MP- related hepatotoxicity was found in 4 patients with NUDT15 CC genotype, and in 2 patients with heterozygous genotype, and no patients homozygous for NUDT15 T allele experienced hepatotoxicity during maintenance therapy (Table 2). Eighteen patients required 6-MP interruption due to severe infection, but the odds ratio was not statistically significant (OR, 0.25; 95% CI, 0.054–1.162).

**Table 2:** Associations between NUDT15 c.415C > T and risk of leukopenia, hepatotoxicity and therapy interruption

	NUDT15 (rs116855232, c.415C > T)			Dominant model
	CC(*n* = 74)	CT(*n* = 29)	TT(*n* = 2)	*P* value OR (95% CI)
Leukopenia (WBC < 2 × 10^9^/L)				
No	63	19	0	0.009
Yes	11	10	2	3.617 (1.377–9.051)
Early-onset leukopenia (WBC < 2 × 10^9^/L)				
No	70	20	0	3.75 × 10^−4^
Yes	4	9	2	9.63(2.764–33.514)
Hepatotoxicity (AST/ALT> 500 IU/L)				
No	70	27	2	0.883
Yes	4	2	0	1.207 (0.209–6.957)
Therapy interruption				
No	58	27	2	0.077
Yes	16	2	0	0.25 (0.054–1.162)

Odds ratios (ORs) and 95% confidence intervals (CI) were calculated using logistic regression

Early-onset leukopenia was defined as leukopenia occurrence during the first 60 days of the maintenance therapy

Dominant model (CT + TT vs CC)

*Abbreviations*: *WBC* white blood cells, *ALT* alanine aminotransferase, *AST* aspartate aminotransferase

## Discussion

The inter-individual genetic variations in drug metabolizing enzymes and transporters influence the efficacy and toxicity of numerous drugs, usually resulting in treatment failure. As a fundamental element in individualized therapy, pharmacogenomics allows for the evaluation of certain genetic variants responsible for drug response and makes better outcome and lower toxicity [30, 31]. TPMT genotyping and its implementation in 6-MP toxicity prediction are considered as a great success in this field. However, differences in the frequency and distribution of TPMT alleles among different ethnic populations limit their predictive value. In this study, we replicated the finding that TPMT*3C is strongly associated with 6-MP tolerance, but more importantly described a NUDT15 c.415C > T variant associated with a substantially elevated risk of 6-MP-associated leukopenia in Chinese children with ALL. There was a significant correlation between the presence of a T allele in the NUDT15 c.415C > T variant and 6-MP dose intensity. Patients with NUDT15 homogenous genotype (TT) were more intolerant to 6-MP (dose intensity of 60.27%), compared with NUDT15 TC and CC carriers. Therefore, NUDT15 genotyping appears to be a better tool for further 6-MP dose optimization and may have great potential for clinical practice.

Although patients with NUDT15 risk allele had a 6-MP dose reduction, the decreased proportion in our study was not consistent with previously reported results [14, 32]. This discrepancy can be attributed to various factors, such as the number of enrolled patients, patient characteristics, variation in the frequency of NUDT15, and differences in 6-MP initial dose and dose adjustment protocol. Among these factors, 6-MP daily dose and dose adjustment protocol are the most important. *Yang* JJ et al. demonstrated that patients who were TT carriers with an initial 6-MP dose at 75 mg/m^2^/d tolerated only 8.3% of the standard 6-MP dose [14]. Moriyama T et al. enrolled 270 ALL patients from Guatemala, Singapore and Japan, and the planned 6-MP dosage was 50–75 mg/m^2^/d with associated risk stratification. The 6-MP dose was adjusted to different WBC counts in the three populations [32]. However, in a recent study from Uruguay [33], the maintenance therapy for childhood ALL, consisting of 6-MP at 50 mg/m^2^ daily and MTX at 20 mg/m^2^ weekly, was the same as that in our protocol. 6-MP dose intensity in individuals with at least one allele for TPMT and/or NUDT15 was approximately 60%, which is in agreement with our study. Thus, additional studies on a larger scale with different 6-MP regimens are warranted to fully elucidate 6-MP tolerance and individualized dose adjustment.

In term of allele frequency, NUDT15 c.415C > T frequency varies among ethnic groups, ranging from 2 to 14%. The allele frequency of NUDT15 in Chinese children with ALL was 15.7%, which was slightly higher than that found in IBD patients (12.1%) [34] and Taiwan Chinese children with ALL (11.6%) [25, 35] in previous studies. Conversely, the NUDT15 risk allele is less common in other populations, with prevalence of 2% in an admixed American population [24], 0.4% in Lebanese [27] and 8.8% in Uruguayan [33]. In contrast to NUDT15, the overall risk allele frequency of TPMT 719A > G was 2.9% in our population. Thus, the clinical value of predicting leukopenia by identifying TPMT genotype in East Asian population was hindered by the low frequency of the risk allele. Additional research in Chinese patients is required to determine the combined influence of both variants on 6-MP-induced toxicity.

ITPA is another important enzyme involved in 6-MP metabolism. We confirmed that the prevalence of ITPA 94 C > A was 18.1% among ALL patients, which is consistent with that reported in other Asian groups. However, genetic polymorphism of ITPA was not correlated with 6-MP dose intensity during maintenance. The results were consistent with these of a recent study in Thai patients with ALL, which also demonstrated a non-association between ITPA variants and 6-MP induced leukopenia and cumulative 6-MP doses at any time point during maintenance [21]. A recent study in Japanese children with ALL also indicated that the ITPA c.94 C > A did not determine the toxicity or the 6-MP dose during maintenance therapy [26]. Therefore, ITPA genotyping is not a predictor for 6-MP induced toxicity in Chinese children with ALL.

Consistent with previous reports [4, 26], we further confirmed the findings that NUDT15 c.415C > T is strongly associated with 6-MP-induced leukopenia in patients with ALL. Notably, NUDT15 c.415C > T was also a strong indicator of early-onset leukopenia. As Yang et al. reported that the NUDT15 variant was strongly associated with early thiopurine-induced leukopenia in a Korean IBD cohort based on GWAS [24]. In a subsequent study, NUDT15 c.415C > T variant was found to be common in Korean patients with various neurological diseases and was strongly associated with azathioprine-induced early leukopenia [36]. As reported in a recent Chinese IBD study, NUDT15 c.415C > T was associated with not only all phases of leukopenia but also early-onset leukopenia [37]. In agreement with these results, our data showed that NUDT15 polymorphism was remarkably associated with leukopenia that developed within the initial 60 days of the maintenance therapy. Although a strong association was observed between NUDT15 genotype, leukopenia, and early-onset leukopenia, the odd ratio was higher for early-onset leukopenia. This finding could be explained by an increased number of individuals with leukopenia without risk alleles due to 6-MP accumulation.

NUDT15, also known as MTH2, is a 164-amino-acid protein that belongs to the nudix hydrolase enzyme family, whose members can hydrolyze compounds with the general structure of a nucleoside diphosphate, such as converting 8-oxo-dGTP and 8-oxo-dGDP to 8-oxo-dGMP [38, 39]. NUDT15 rs116855232 is located in exon 3, causing an arginine-to-cysteine (p.Arg139Cys) mutation that consequently leads to changes in the amino acid sequence of the NUDT15 protein [24]. The impacts of NUDT15 rs116855232 on thiopurines induced myelotoxicity and on thiopurines intolerance are well established and have been replicated recently in studies of patients with IBD [24, 34, 40–42] and ALL [14, 19, 21, 25, 32]. Subsequently, three more coding variants (p.Arg139His, p.Val18Ile, and p.Val18_Val19insGlyVal) and six haplotypes (*1–*6) have been identified [32, 43]. Additional NUDT15 diplotypic groups were established according to enzymatic activity. However, the mechanism underlying the NUDT15 -related leukopenia remains unknown. As reported by Moriyama T et al. [32], NUDT15 inactivates thiopurine metabolites and decreases thiopurine cytotoxicity in vitro, and patients with NUDT15 risk alleles have excessive levels of active thiopurine metabolites, leading to an increase in mercaptopurine-induced toxicity. Of note, a recent study demonstrated that the loss of NUDT15 had no effect on the incorporation of 8-oxo-dGTP, and NUDT15 processes other nucleotide substrates over 8-oxo-dGTP [44]. These results suggest an additional function of NUDT15 in physiological status and that other mechanism are involved in thiopurine metabolite production. It is unclear how the coding variant influences NUDT15 enzymatic activity by affecting NUDT15 protein synthesis and/or degradation. Intriguingly, a new study revealed that NUDT15 p.Arg139Cys mutation failed to affect enzymatic activity, but negatively influenced protein stability, possibly due to a loss of supportive intramolecular bonds that caused a rapid proteasomal degradation in cells [45].

Our study has some limitations. First, this study has a small sample size, resulting in a low power to detect differences between small subsets. Second, we failed to analyze the clinical outcomes associated with NUDT15 and other genotypes. Moreover, the mechanism underlying the NUDT15 variant leading to leukopenia was not clarified, including the activity of the enzyme, and how the variant influences the toxicity of 6-MP. Further studies with more patients are needed to clarify these questions.

## Conclusions

We elucidated the frequency of NUDT15, TPMT, and ITPA polymorphisms in Chinese children with ALL. Our data strongly support the predictive role of NUDT15 rs116855232 in 6-MP intolerance and hematopoietic toxicity. Further clinical studies are thus required to evaluate the importance of upfront NUDT15 genotyping to develop better and more rational treatment strategies in ALL patients.

## Abbreviations

6-MP: 6-mercaptopurine
ALL: Acute lymphoblastic leukemia
DEX: Dexamethasone
GWAS: Genome-wide association study
IBD: Inflammatory bowel disease
ITPA: Inosine triphosphate pyrophosphohydrolase
MTX: Methotrexate
NUDT15: Nucleoside diphosphate-linked moiety X motif 15
TPMT: Thiopurine S-methyltransferase
VCR: Vincristine
WBC: White blood cell
